# Supplementary material for: In Vivo Evaluation of DMSA-Coated Magnetic Nanoparticle Toxicity and Biodistribution in Rats: A Long-Term Follow-Up
Source: Nanomaterials (Basel). 2022 Oct 8;12(19):3513. doi: 10.3390/nano12193513 (PMC9565739; doi:10.3390/nano12193513)
Supplement: Supplementary file 1 [file nanomaterials-12-03513-s001.zip › nanomaterials-1934196-supplementary.pdf]

Supplementary Table S1. Hematological parameters from Control group (injection of 100µL saline solution) throughout 300 days. Data are mean ± SD (minimum – maximum values)

Parameter	**D0**	**D15**	**D30**	**D60**	**D90**	**D120**	**D150**	**D180**	**D210**	**D240**	**D270**	**D300**
RBC ($x10^6/\mu L$)	6.7±0.5 (6.2-7.4)	10.8±1.5 (10.2-11.8)	7.8±0.3 (7.3-8.1)	8.1±0.2 (7.7-8.3)	8.4±0.3 (8.1-8.8)	7.8±0.2 (7.4-8.0)	8.6±1.1 (7.5-10.4)	8.7±1.2 (8.9-9.7)	8.6±0.7 (7.9-9.5)	8.5±0.5 (7.7-9.2)	9.5±1.1 (8.1-10.8)	9.2±1.3 (7.8-10.9)
HB (g/dL)	14.1±1.2 (12.5-15.5)	22.2±3.3 (20.8-24.7)	15.7±0.7 (14.6-16.7)	16.0±0.5 (15.5-16.6)	16.7±0.5 (16.0-17.2)	15.4±0.5 (14.7-16.0)	16.6±2.4 (14.1-20.6)	17.0±2.3 (13.0-18.6)	16.9±1.8 (15.0-19.6)	17.0±1.1 (15.6-18.4)	18.6±2.1 (16.2-20.8)	18.0±2.7 (15.3-21.8)
HTC (%)	37.2±3.5 (33.1-43.0)	60.2±8.8 (43.6-66.5)	44.1±1.7 (41.9-45.8)	43.9±0.8 (43.0-44.8)	44.6±1.9 (42.1-46.6)	40.8±1.8 (38.4-43.0)	45.3±5.9 (39.7-55.3)	45.4±6.1 (34.7-50.3)	45.1±4.5 (40.9-51.6)	44.9±2.2 (42.7-48.5)	50.4±5.8 (43.2-56.9)	49.5±7.6 (41.4-60.0)
MCV (fL)	55.4±1.5 (53.6-58.0)	55.9±0.7 (54.9-56.6)	56.1±0.6 (55.2-57.1)	54.1±1.2 (52.5-56.0)	53.1±0.7 (51.9-53.6)	52.3±0.8 (51.6-53.5)	52.8±0.5 (52.2-53.3)	52.1±0.7 (51.5-53.4)	52.4±1.0 (51.5-54.1)	52.7±1.3 (51.3-54.7)	53.1±0.5 (52.5-53.5)	53.9±0.8 (53.4-55.3)
MCH ($x10^3/\mu L$)	21.0±0.4 (20.3-21.4)	20.6±0.2 (20.4-20.9)	20.0±0.3 (19.8-20.5)	19.8±0.3 (19.5-20.0)	19.9±0.3 (19.6-20.3)	19.8±0.1 (19.7-19.9)	19.3±0.5 (18.8-19.8)	19.6±0.6 (19.0-20.5)	19.6±0.6 (18.9-20.5)	19.9±0.3 (19.5-20.3)	19.6±0.5 (18.9-20.1)	19.6±0.3 (19.4-20.1)
MCHC (g/dL)	37.8±1.0 (36.0-38.7)	37.0±0.5 (36.4-37.9)	35.7±0.6 (34.8-36.5)	36.6±1.0 (35.7-38.2)	37.4±0.6 (36.7-38.0)	37.8±0.5 (37.2-38.3)	36.6±0.7 (35.5-37.3)	37.5±0.6 (36.9-38.4)	37.5±0.7 (36.7-38.4)	37.6±1.0 (36.4-38.5)	36.9±0.6 (36.0-37.5)	36.4±0.5 (35.8-37.0)
PLT ($x10^3/\mu L$)	659±318 (129-1002)	446±190 (299-817)	845±69 (762-948)	788±211 (433-977)	752±181 (454-937)	693±209 (323-826)	464±271 (184-832)	495±233 (148-742)	799±164 (618-1055)	839±88 (718-934)	613±160 (352-791)	661±151 (437-813)
WBC ($x10^3/\mu L$)	7.0±3.0 (4.7-12.0)	8.9±2.1 (6.2-12.2)	10.7±1.6 (8.2-12.7)	8.9±1.3 (7.7-10.7)	8.3±0.4 (8.0-8.8)	5.7±0.6 (4.8-6.6)	4.6±1.2 (3.3-6.6)	5.1±0.8 (3.6-5.5)	6.1±1.5 (4.8-8.6)	5.5±1.0 (4.4-6.9)	5.6±0.5 (4.9-6.1)	3.2±0.5 (2.4-8.6)
LP (%)	76.3±6.1 (67.8-80.8)	70.1±3.9 (64.4-74.9)	74.1±3.8 (68.5-79.7)	76.0±2.1 (73.0-78.6)	70.4±2.5 (66.3-73.0)	66.5±7.4 (53.8-73.2)	70.4±5.4 (66.3-75.7)	68.8±1.9 (66.4-71.7)	63.1±8.5 (48.9-70.7)	65.2±2.9 (61.3-67.9)	65.0±2.1 (61.6-67.0)	61.3±5.5 (53.6-67.4)
MP (%)	23.7±6.1 (17.5-32.2)	29.9±3.9 (25.1-33.1)	25.9±3.8 (20.3-27.8)	24.0±2.1 (21.4-27.0)	29.6 ±2.5 (27.0-33.7)	33.5±7.4 (26.8-46.2)	29.6±5.4 (24.3-36.7)	31.2±1.9 (28.3-33.6)	36.9±8.5 (29.3-51.7)	34.8±2.9 (32.1-38.7)	35.0±2.1 (33.0-38.4)	38.7±5.5 (32.6-46.4)
LN ($x10^3/\mu L$)	5.3±2.1 (3.3-8.4)	6.3±1.7 (4.4-9.1)	8.0±1.3 (5.6-9.3)	6.8±1.1 (5.9-8.3)	5.9±0.2 (5.7-6.1)	3.8±0.7 (3.1-4.8)	3.2±0.9 (2.4-4.8)	3.5±0.6 (2.5-3.9)	3.8±1.0 (3.0-5.3)	3.6±0.5 (2.9-4.2)	3.6±0.3 (3.3-4.0)	1.9±0.3 (1.5-2.2)
MN ($x10^3/\mu L$)	1.7±1.0 (0.8-3.6)	2.7±0.6 (1.8-3.6)	2.8±0.6 (2.0-3.4)	2.1±0.3 (1.8-2.4)	2.5 ±0.3 (2.2-3.0)	1.9±0.4 (1.6-2.6)	1.4±0.4 (0.9-1.8)	1.6±0.3 (1.1-1.8)	2.3±0.8 (1.6-3.3)	1.9±0.5 (1.5-2.7)	2.0±0.2 (1.6-2.2)	1.3±0.3 (0.9-1.7)

WBC: white blood cell count, RBC: red blood cell count, HB: hemoglobin concentration, HTC: hematocrit, MCV: mean corpuscular volume, MCH: mean corpuscular hemoglobin, MCHC: mean cell hemoglobin concentration, PLT: platelet, LP: lymphocyte percent, MP: monocyte percent, LN: lymphocyte number, MN: monocyte number.

Supplementary Table S2. Hematological parameters from T0.5 group (injection of 100µL DMSA-MNP solution at 0.5 mg Fe/kg of body weight) throughout 300 days. Data are mean ± SD (minimum – maximum values)

Parameter	**D0**	**D15**	**D30**	**D60**	**D90**	**D120**	**D150**	**D180**	**D210**	**D240**	**D270**	**D300**
***RBC* (*x*$10^6/\mu L$)**	7.1±0.7 (6.1-7.8)	8.2±1.8 (5.9-10.3)	8.3±0.4 (7.8-8.7)	8.5±0.7 (8.1-9.4)	8.2±0.4 (7.6-8.7)	7.7±0.4 (7.2-8.4)	8.0±0.5 (7.4-8.6)	8.9±1.3 (7.5-10.8)	7.6±1.3 (5.3-8.4)	7.9±0.6 (7.3-8.4)	9.0±0.4 (8.8-9.6)	8.5±0.7 (7.7-9.3)
***HB* (*g/dL*)**	15.1±1.4 (12.4-16.6)	17.5±3.7 (12.4-22.3)	17.2±1.1 (16.9-18.1)	17.4±1.5 (16.0-18.4)	16.7±0.8 (15.8-17.6)	15.7±0.8 (14.4-16.7)	16.3±1.0 (15.2-17.6)	17.7±2.7 (15.2-21.5)	15.3±1.3 (11.1-16.8)	16.1±0.9 (15.0-16.9)	18.4±0.3 (18.0-18.8)	17.1±1.2 (16.0-18.6)
***HTC* (*%*)**	39.9 ±3.6 (33.1-43.8)	46.8±9.7 (33.9-59.5)	46.9±2.4 (43.3-49.0)	46.7±3.7 (43.4-49.3)	44.7±2.3 (42.3-44.8)	42.0±1.9 (39.2-44.4)	43.5±2.4 (40.8-46.3)	47.5±6.8 (41.0-56.5)	40.9±6.9 (28.6-44.8)	42.0±3.2 (38.0-47.4)	49.4±1.6 (47.8-51.5)	47.1±3.9 (43.1-51.4)
***MCV* (*fL*)**	56.4±2.0 (53.9-59.7)	57.0±1.3 (55.3-58.7)	56.8±1.1 (56.0-58.0)	54.8±0.7 (54.0-55.8)	54.5±0.8 (53.6-55.6)	54.4±1.0 (53.1-54.7)	54.0±0.8 (53.3-55.4)	53.7±1.1 (52.6-54.7)	53.7±0.4 (53.1-54.1)	54.3±1.5 (52.2-55.4)	54.7±1.2 (53.6-55.9)	55.3±0.7 (54.4-56.1)
***MCH* (*x*$10^3/\mu L$)**	21.4±0.7 (20.2-22.5)	20.0±3.5 (12.2-22.6)	20.8±0.6 (19.7-21.2)	20.4±0.3 (20.1-20.7)	20.4±0.4 (20.0-21.0)	20.3±0.4 (20.0-21.0)	20.3±0.4 (19.8-20.7)	20.0±0.2 (19.6-20.3)	20.2±0.4 (19.8-20.8)	20.3±0.2 (20.1-20.6)	20.3±0.5 (19.6-20.8)	20.1±0.5 (19.7-20.8)
***MCHC* (*g/dL*)**	37.9±0.7 (37.5-38.6)	37.4±0.8 (36.3-38.6)	35.6±0.7 (36.3-37.2)	37.2±0.3 (36.9-37.6)	37.5±0.6 (36.7-38.4)	37.3±0.5 (36.7-37.9)	37.5±0.4 (37.1-38.0)	37.2±0.5 (36.7-38.1)	37.6±0.7 (36.9-38.8)	37.4±0.9 (36.4-38.5)	37.2±0.5 (36.5-37.7)	36.4±0.6 (35.7-37.1)
***PLT* (*x*$10^3/\mu L$)**	720±414 (140-1123)	463±325 (73-858)	807±215 (651-1167)	638±248 (211-822)	505±150 (377-765)	551±376 (41-920)	811±86 (723-905)	421±279 (123-744)	622±397 (116-992)	550±468 (19-1065)	611±242 (261-776)	579±88 (455-646)
***WBC* (*x*$10^3/\mu L$)**	6.2±2.5 (3.9-10.6)	8.9±2.0 (6.1-11.7)	10.3±2.2 (8.1-13.5)	9.3±1.8 (6.8-11.1)	7.1±1.7 (4.8-9.4)	5.2±2.1 (3.4-8.3)	5.2±1.2 (4.1-7.0)	4.9±0.2 (4.0-7.0)	5.4±1.1 (4.1-6.4)	6.1±0.2 (5.9-6.3)	4.9±1.6 (3.2-6.9)	2.5±1.0 (1.8-3.9)
***LP* (*%*)**	74.9±6.7 (60.4-79.7)	72.5±6.1 (65.1-79.5)	73.7±3.7 (67.5-76.7)	72.7±2.4 (69.1-74.7)	70.4±1 (69.3-71.8)	65.8±4.5 (61.0-71.0)	65.7±4.8 (58.0-70.2)	66.0±4.6 (58.7-69.6)	67.9±6.3 (60.7-76.7)	68.0±8.5 (56.2-76.4)	61.0±7.1 (50.8-66.3)	59.3±6.3 (53.0-66.7)
***MP* (*%*)**	25.1±6.7 (20.3-39.6)	27.5±6.1 (23.8-34.9)	26.3±3.7 (23.3-32.5)	27.3±2.4 (24.9-28.2)	29.6±1.0 (28.2-30.7)	34.2±4.5 (29.0-37.7)	34.3±4.8 (29.8-42.0)	34.0±4.5 (30.4-41.3)	32.1±6.3 (23.3-39.3)	32.1±8.5 (23.6-43.8)	39.0±7.1 (33.7-49.2)	40.7±6.3 (33.3-47.0)
***LN* (*x*$10^3/\mu L$)**	4.7±2.0 (2.4-8.2)	6.5±1.7 (4.0-9.2)	7.6±1.5 (6.4-9.9)	6.8±1.4 (4.7-8.3)	5.0±1.1 (3.4-6.5)	3.4±1.4 (2.1-5.2)	3.5±1.0 (2.4-4.8)	3.3±0.9 (2.3-4.8)	3.7±0.7 (2.7-4.3)	3.7±1.1 (2.4-4.8)	3.0±1.2 (1.6-4.5)	1.5±0.7 (1.0-2.6)
***MN* (*x*$10^3/\mu L$)**	1.5±0.6 (0.9-2.4)	2.4±0.6 (1.6-3.4)	2.7±0.8 (2.0-3.6)	2.5±0.4 (2.0-2.8)	2.1±0.6 (1.4-2.9)	1.8±0.8 (1.2-3.1)	1.7±0.3 (1.4-2.2)	1.7±0.4 (1.2-2.2)	1.8±0.5 (1.0-2.4)	1.7±0.7 (1.0-2.6)	1.9±0.5 (1.3-2.4)	1.0±0.3 (0.7-1.3)

WBC: white blood cell count, RBC: red blood cell count, HB: hemoglobin concentration, HTC: hematocrit, MCV: mean corpuscular volume, MCH: mean corpuscular hemoglobin, MCHC: mean cell hemoglobin concentration, PLT: platelet, LP: lymphocyte percent, MP: monocyte percent, LN: lymphocyte number, MN: monocyte number.

Supplementary Table S3. Hematological parameters from T5 group (injection of 100µL DMSA-MNP solution at 5 mg Fe/kg of body weight) throughout 300 days. Data are mean ± SD (minimum – maximum values)

Parameter	D0	D15	D30	D60	D90	D120	D150	D180	D210	D240	D270	D300
RBC (x10^6/µL)	10.0±0.7 (9.3-10.8)	8.5±1.2 (6.7-9.3)	8.9±1.2 (7.5-10.6)	8.2±0.3 (7.9-8.5)	7.5±1.7 (4.6-8.5)	8.3±0.7 (7.9-9.6)	7.9±0.5 (7.0-8.2)	8.3±0.4 (7.8-8.8)	8.8±0.6 (8.2-9.7)	8.0±0.6 (7.0-8.5)	7.9±0.5 (7.3-8.5)	7.8±0.3 (7.7-10.9)
HB (g/dL)	21.6±1.5 (19.9-23.5)	18.3±2.5 (14.2-20.4)	18.5±2.3 (15.9-21.5)	16.6±0.3 (16.2-16.9)	15.2±3.5 (9.0-17.4)	16.6±1.1 (15.9-18.5)	15.9±1.2 (13.9-16.7)	16.6±0.7 (16.2-17.3)	17.8±1.5 (16.6-20.0)	16.3±1.6 (13.8-17.9)	15.8±1.0 (14.4-16.6)	15.6±0.8 (15.3-21.8)
HTC (%)	55.9±3.4 (51.5-60.1)	48.3±6.2 (38.4-53.1)	50.6±6.0 (44.4-58.8)	45.8±1.0 (44.5-46.1)	41.2±8.9 (25.4-46.3)	45.2±3.3 (43.2-51.1)	43.0±2.8 (38.8-45.3)	44.5±2.0 (41.6-46.6)	47.7±3.9 (44.2-53.4)	42.7±4.0 (36.4-46.7)	42.7±2.9 (39.1-45.1)	42.9±2.0 (41.1-60.0)
MCV (fL)	56.0±1.1 (54.8-57.5)	56.5±0.9 (55.3-57.1)	57.0±1.3 (55.5-58.7)	55.7±0.9 (54.4-56.3)	54.6±0.9 (53.7-55.8)	54.3±0.6 (53.3-54.7)	54.3±1.1 (52.5-54.7)	53.5±1.0 (52.2-54.8)	53.9±0.7 (53.3-55.1)	53.6±1.1 (52.2-55.2)	54.3±1.4 (52.9-56.4)	49.1±0.1 (53.4-57.0)
MCH (x10^3/µL)	21.6±0.3 (21.2-22.0)	21.3±0.5 (20.8-21.9)	20.8±0.4 (20.3-21.3)	20.1±0.4 (19.8-20.6)	20.1±0.4 (19.5-20.7)	19.9±0.4 (19.3-20.3)	20.1±0.3 (19.8-20.5)	20.0±0.4 (19.5-20.5)	20.1±0.4 (19.6-20.6)	20.4±0.6 (19.8-21.2)	20.1±0.7 (19.3-21.1)	17.9±0.1 (19.4-20.8)
MCHC (g/dL)	38.7±0.4 (38.1-39.1)	37.8±0.6 (37.0-38.4)	36.5±0.5 (35.8-37.0)	36.2±0.3 (35.9-36.6)	36.8±0.8 (35.4-37.6)	36.7±0.4 (36.2-37.2)	36.9±0.6 (36.2-37.6)	37.4±0.2 (37.1-37.4)	37.4±0.4 (36.8-37.8)	38.1±0.3 (37.3-38.4)	37.1±0.4 (36.6-37.6)	32.4±0.1 (35.7-37.1)
PLT (x10^3/µL)	673±31 (628-714)	531±195 (298-702)	608±289 (130-871)	625±332 (76-849)	552±362 (128-903)	655±328 (94-884)	504±337 (204-928)	586±362 (27-877)	748±130 (581-916)	702±285 (229-942)	540±319 (200-892)	667±190 (337-795)
WBC (x10^3/µL)	6.4±1.4 (4.5-8.3)	7.9±0.8 (6.8-8.7)	10.5±4.4 (5.2-15.5)	6.3±2.0 (4.7-9.0)	6.4±2.7 (2.3-8.7)	5.3±1.3 (4.5-7.5)	4.1±1.4 (2.3-5.6)	3.7±1.2 (2.9-5.8)	4.3±1.0 (3.5-6.1)	4.5±1.9 (2.9-7.6)	4.2±1.0 (3.3-5.9)	2.5±0.2 (1.8-4.1)
LP (%)	71.3±2.6 (68.5-75.0)	65.0 ±8.2 (53.2-71.2)	71.5±11.8 (59.1-90.8)	68.1±6.0 (59.8-76.5)	67.4±2.7 (65.2-71.6)	64.0±6.8 (56.3-74.8)	59.1±6.6 (50.5-66.3)	64.3±5.2 (56.9-69.7)	59.2±4.1 (56.5-66.3)	62.4±4.9 (56.8-69.1)	59.7±7.9 (48.5-68.0)	53.6±0.6 (51.3-68.7)
MP (%)	28.7±2.6 (25.0-31.5)	34.9±8.2 (28.5-46.8)	28.5±11.8 (9.2-40.9)	31.9±6.0 (23.5-40.2)	32.6±2.7 (28.4-34.8)	36.0±6.8 (25.2-43.7)	40.9±6.6 (33.7-49.5)	35.7±5.2 (30.3-43.1)	40.8±4.1 (33.7-43.5)	37.6±4.9 (30.9-43.2)	40.3±7.9 (32.0-51.5)	36.6±0.7 (31.1-48.7)
LN (x10^3/µL)	4.6±1.0 (3.4-6.0)	5.2±1.1 (3.9-6.2)	7.2±2.3 (4.7-10.0)	4.4±1.7 (3.2-6.3)	4.3±1.8 (1.5-5.7)	3.5±1.2 (2.5-5.6)	2.4±0.8 (1.5-3.6)	2.4±0.9 (1.8-3.9)	2.5±0.6 (2.0-3.4)	2.9±1.3 (1.6-4.9)	2.5±0.9 (1.8-4.0)	1.5±0.2 (1.0-2.8)
MN (x10^3/µL)	1.8±0.5 (1.1-2.3)	2.7±0.4 (2.5-3.4)	3.3±2.1 (0.5-5.5)	2.0±0.5 (1.5-2.7)	2.1±0.9 (0.8-3.0)	1.9±0.2 (1.7-2.0)	1.7±0.7 (0.8-2.8)	1.3±0.4 (0.9-1.9)	1.8±0.5 (1.4-2.7)	1.7±0.6 (1.3-2.7)	1.6±0.3 (1.2-2.0)	1.0±0.1 (0.7-1.7)

WBC: white blood cell count, RBC: red blood cell count, HB: hemoglobin concentration, HTC: hematocrit, MCV: mean corpuscular volume, MCH: mean corpuscular hemoglobin, MCHC: mean cell hemoglobin concentration, PLT: platelet, LP: lymphocyte percent, MP: monocyte percent, LN: lymphocyte number, MN: monocyte number.

Supplementary Table S4. Blood biochemical parameters from Control group (injection of 100µL saline solution) throughout 300 days. Data are mean ± SD (minimum – maximum values)

Parameter	D0	D15	D30	D60	D90	D120	D150	D180	D210	D240	D270	D300
Creatinine K (mg/dL)	0.6±0.1 (0.5-0.7)	0.7±0.1 (0.6-0.9)	0.9±0.3 (0.8-1.4)	1.1±0.6 (0.8-2.1)	0.9±0.1 (0.8-0.9)	0.5±0.1 (0.4-0.7)	0.7±0.4 (0.5-1.5)	0.5±0.1 (0.5-0.6)	0.7±0.1 (0.7-0.8)	0.7±0.1 (0.6-0.8)	0.8±0.3 (0.6-1.1)	0.3±0.3 (0.1-0.7)
Ureia (mg/dL)	67.3±17.3 (51-95)	80.8±9.8 (69-94)	94.7±9.1 (78-103)	80.8±8.0 (73-89)	80.0±6.0 (74-89)	56.4±10.9 (39-69)	38.6±13.2 (21-58)	51.6±6.5 (47-63)	45.6±4.8 (38-50)	44.5±3.1 (42-48)	39.8±4.1 (33-44)	39.8±10.1 (23-50)
ALT (U/L)	59.2±19.1 (42-90)	83.0±24.0 (53-126)	90.8±18.7 (68-117)	85.0±50.6 (46-173)	89.2±32.9 (35-125)	46.4±25.5 (19-80)	44.8±14.9 (21-60)	53.2±23.9 (14-71)	36.2±14.7 (18-56)	51.0±28.2 (18-89)	51.2±23.5 (29-89)	42.8±22.8 (10-71)
AST (U/L)	204.7±64.5 (164-333)	218.5±51.0 (170-288)	263.3±84.9 (173-379)	233.4±85.0 (153-364)	237.0±41.8 (166-272)	186.0±53.3 (130-243)	154.4±133.9 (84-393)	158.2±63.5 (62-230)	173.6±39.7 (112-217)	154.8±34.3 (127-210)	145.8±44.7 (92-195)	192.2±50.3 (118-232)
Iron (µg/dL)	254.3±56.0 (184-321)	314.0±26.9 (283-328)	394.6±43.8 (317-420)	308.6±73.7 (191-379)	309.4±60.5 (249-403)	263.0±109.5 (140-434)	211.0±98.3 (41-280)	262.6±44.3 (191-306)	295.4±42.7 (250-354)	286.8±51.5 (250-375)	371.4±54.9 (288-434)	296.6±131.5 (142-478)

Supplementary Table S5. Blood biochemical parameters from T0.5 group (injection of 100µL DMSA-MNP solution at 0.5 mg Fe/kg of body weight) throughout 300 days. Data are mean ± SD (minimum – maximum values)

Parameter	D0	D15	D30	D60	D90	D120	D150	D180	D210	D240	D270	D300
Creatinine K (mg/dL)	0.7±0.1 (0.5-0.9)	0.7±0.1 (0.6-0.7)	0.7±0.1 (0.5-0.8)	0.8±0.1 (0.8-0.9)	0.7±0.2 (0.4-0.9)	0.4±0.2 (0.1-0.6)	0.6±0.1 (0.5-0.7)	0.7±0.1 (0.6-0.8)	0.8±0.1 (0.7-1.0)	0.8±0.2 (0.5-1.0)	0.9±0.1 (0.8-0.9)	0.5±0.1 (0.4-0.6)
Ureia (mg/dL)	66.3±10.8 (51-80)	76.1±7.2 (66-84)	66.0±30.3 (15-87)	80.4±4.3 (75-86)	73.6±15.4 (53-85)	52.2±13.7 (36-70)	46.0±0.6 (39-54)	57.6±4.3 (53-63)	52.0±3.6 (48-55)	52.8±8.8 (39-61)	41.5±3.5 (38-45)	42.0±12.0 (28-57)
ALT (U/L)	49.9±19.5 (28-72)	79.6±16.5 (65-112)	78.8±35.1 (51-125)	74.0±7.6 (66-84)	89.6±34.3 (58-136)	70.8±36.6 (51-136)	51.4±8.0 (40-61)	58.6±10.0 (44-69)	59.7±2.9 (58-63)	60.2±11.6 (43-71)	63.0±22.3 (43-95)	52.5±14.6 (35-65)
AST (U/L)	192.3±57.8 (127-290)	147.2±16.8 (124-174)	238.0±107.7 (136-336)	146.4±71.3 (51-251)	146.8±54.6 (105-227)	201.2±140.8 (106-445)	113.4±32.9 (75-156)	164.2±39.0 (128-226)	175.0±128.5 (47-304)	213.4±111.0 (122-374)	139.0±25.4 (113-165)	201.3±52.2 (133-260)
Iron (µg/dL)	320.3±123.8 (222-556)	349.3±39.7 (294-396)	319.5±268.0 (130-509)	347.0±30.1 (317-396)	318.2±37.2 (273-372)	236.4±40.9 (167-273)	277.0±43.9 (239-348)	280.4±39.9 (223-333)	308.4±22.7 (306-337)	240.2±49.9 (163-295)	337.2±177.2 (181-667)	343.5±103.0 (235-474)

Supplementary Table S6. Blood biochemical parameters from T5 group (injection of 100µL DMSA-MNP solution at 5 mg Fe/kg of body weight) throughout 300 days. Data are mean ± SD (minimum – maximum values)

Parameter	D0	D15	D30	D60	D90	D120	D150	D180	D210	D240	D270	D300
Creatinine K (mg/dL)	0.8±0.1 (0.6-1.0)	0.7±0.1 (0.6-0.8)	0.8±0.1 (0.7-0.9)	0.8±0 (0.8-0.9)	0.9±0.2 (0.7-1.2)	0.5±0.1 (0.5-0.6)	0.4±0.4 (0.1-0.8)	0.6±0.1 (0.5-0.7)	0.8±0.2 (1.3-1.6)	0.8±0.1 (0.7-0.9)	0.9±0.1 (0.7-1.0)	0.5±0.4 (0.1-0.9)
Ureia (mg/dL)	82.2±8.8 (70-94)	76.4±12.3 (63-92)	102.8±7.9 (93-107)	79.8±5.1 (74-85)	83.8±5.9 (77-90)	71.0±4.8 (65-78)	52.8±13.3 (36-68)	53.8±4.3 (49-60)	50.4±4.7 (43-56)	47.6±6.4 (44-59)	46.4±5.7 (38-54)	49.2±9.3 (43-64)
ALT (U/L)	54.2±15.1 (37-73)	76.6±17.9 (62-107)	69.8±30.7 (26-91)	65.3±14.5 (53-86)	88.4±17.4 (68-108)	67.2±11.5 (56-85)	42.8±16.5 (20-63)	55.6±75.0 (49-66)	51.6±13.1 (40-73)	60.4±14.5 (53-80)	63.0±21.0 (42-98)	62.2±37.8 (39-129)
AST (U/L)	188.5±60.1 (144-277)	142.8±29.7 (109-178)	305.5±48.8 (271-340)	206.8±114.9 (137-378)	206.8±43.6 (162-266)	197.4±64.4 (119-271)	137.6±52.3 (78-212)	176.0±54.8 (116-266)	121.0±38.4 (85-174)	129.8±40.7 (101-201)	151.8±60.5 (94-249)	185.4±49.9 (126-226)
Iron (µg/dL)	310.5±95.0 (225-423)	479.3±97.6 (376-570)	403.0±38.2 (376-430)	345.5±42.2 (294-386)	328.8±30.9 (292-365)	254.6±14.8 (239-270)	260.0±48.6 (201-331)	271.4±38.6 (212-310)	298.6±36.2 (254-347)	348.6±85.5 (243-465)	376.6±87.4 (299-507)	303.8±139.0 (205-542)
